# Gender differences in operational and cognitive abilities

**DOI:** 10.3389/fpsyg.2024.1402645

**Published:** 2024-10-22

**Authors:** Emil Lager, Kimmo Sorjonen, Marika Melin

**Affiliations:** Department of Clinical Neuroscience, Karolinska Institutet, Stockholm, Sweden

**Keywords:** cognitive abilities, gender differences, aviation, multitasking, spatial ability

## Abstract

**Introduction:**

Gender differences in cognitive and operational abilities have been identified. Yet, their interrelationship remains underexplored. This prevents tailored evidence-based selection, allowing discrimination to persist.

**Methods:**

Data from a test battery of operational and cognitive tests was analyzed. In total 2,743 aviation pilot candidates’ test scores were analyzed.

**Results:**

Males had a significantly higher score on mental spatial ability, memory retention, abstract problem solving, multitasking ability (MU), and manual spatial ability (MSA); and females on perceptual speed. Correlations between MU and MSA [difference = 0.269 (95% CI: 0.114; 0.405)] and between MSA and perceptual speed [difference = 0.186 (95% CI: 0.027; 0.332)] were significantly stronger among female applicants. A high MSA score was more predictive of a high score on MU, Perceptual speed, and Memory for female compared with male applicants (*p* < 0.002 for the MSA score × sex interaction effect in all three cases).

**Discussion:**

Interpretation of test scores in between genders potentially may need to look different for final selection decisions for operational professions, as female test profiles were shown to exhibit greater homogeneity.

## 1 Introduction

The impact of child gender differences is a question which has received broad attention in research (e.g., Geary, 2010). General intelligence explains a large portion of intraindividual differences in a multitude of non-trivial outcomes (e.g., Gottfredson, 1998, 2004; Plomin and Deary, 2015; Ree and Carretta, 2022). The debate on potential gender differences in general intelligence has been approached from several angles. As Hunt (2010), describes, evidence on general intelligence comes from three types of sources: (i) studies based on single tests (e.g., Raven Matrices); (ii); studies on the g-factor (e.g., Deary and Batty, 2007; Gottfredson, 1998); (iii) and studies on overall scores from test batteries like the Weschler Scales. Put together, the research body is somewhat inconclusive regarding gender differences in general intelligence. Some studies have found that females outperform males (e.g., Arden and Plomin, 2006; Reynolds et al., 2008). Many studies have found the inverse (e.g., Flores-Mendoza et al., 2013; Jackson and Rushton, 2006; Lynn and Irwing, 2004). Crucially, methodological issues and non-representative data-samples further blurs the picture, as problematized by Savage-McGlynn (2012). Other findings are indicative of no significant difference between the genders in terms of mean general intelligence (e.g., Halpern and LaMay, 2000; Neisser et al., 1996). According to Nowell and Hedges (1998), gender differences in cognitive tests are small in terms of means and variance– and this has been the case since 1960– while differences in extreme scores often can be substantial. In line with that, Lubinski and Humphreys (1990) found that in terms of variance within the genders, men have been found among the lowest and highest scorers, with a 7 percent larger standard deviation for males.

However, in specific components of cognitive ability (general cognitive ability and general intelligence are used synonymously), gender differences have repeatedly been reported (e.g., Boyle et al., 2010; Halpern and LaMay, 2000). Numerous studies have identified differences in favor of males in different types of visuospatial abilities involving mental rotation (e.g., Halpern, 2013; Halpern and LaMay, 2000; Neubauer et al., 2005; Voyer et al., 1995). These types of tasks often yield the most consistent and largest gender differences, favoring males (see for instance, Linn and Petersen, 1985; Voyer et al., 1995). However– while most findings suggest a robust gender difference favoring males in visuospatial tasks– as noted by Kheloui et al. (2023); different methodologies yield different conclusions about the magnitude of the gender difference. Males have also been found to outperform females in some numerical tasks (Guiso et al., 2008; Weber et al., 2014). In mathematical achievement, large effects favoring males have been identified, especially when the sample is highly select (Halpern and LaMay, 2000).

Females tend to outperform males in tests of verbal abilities and reading comprehension (Guiso et al., 2008; Halpern and LaMay, 2000; Neubauer et al., 2005). The differences found in meta-analyses on verbal abilities is mainly small (Hirnstein et al., 2023; Petersen, 2018). According to Halpern and LaMay (2000), females demonstrate an advantage in abilities making use of verbal information in tasks that require retrieval from long-term memory. Further research has identified that females have outperformed males in tests of episodic memory (Herlitz and Rehnman, 2008). A meta-analysis of 617 studies with over 1 million participants, showed a female episodic memory advantage of Hedge’s g = −0.19, 95% CI [0.17, 0.21](Asperholm et al., 2019). Different tasks yielded different effect sizes, for instance: verbal material (*g* = −0.28, 95% CI [0.25, 0.30]) and routes (*g* = 0.24, 95% CI [−0.35, −0.12]). Thus, the direction and size of a gender difference seems to be task/stimulus dependent.

With respect to operational abilities, males have been found to outperform females in navigational skills (Wang et al., 2020). Some recent findings show no gender differences in several different versions of multitasking tests, even when controlling for gender differences in underlying cognitive abilities (Hirsch et al., 2019). There are also findings showing that differences in multitasking ability do exist– with men performing better– and that the differences are mediated by spatial ability (Mäntylä, 2013).

It has been argued that evidence-based practices in psychology could be as stringently applied in assessment, as they are in treatment (see for instance, (Bornstein, 2017). Selection for many high-risk jobs involves a combination of cognitive and operational tests. However, the interrelationship between different cognitive and operational abilities– especially from a gender perspective– has received very little attention in research. In aviation, selecting adequately skilled pilots is crucial for safe and effective flight operations. In recent years, the aviation industry has faced a growing challenge concerning pilot supply and the quality of pilots, Thus, a substantial pilot shortage is expected (Caraway, 2020; Klapper and Ruff-Stahl, 2019). Aviation is a sector traditionally staffed by men and gender segregation persists (Gagliardo, 2020; Mills et al., 2014). The imminent shortage is not softened to the extent it could be if more women entered the workforce. In an Air Force pilot sample, an examination of [general] intelligence assessed through the adult Weschler scale, demonstrated no real differences between male and female pilots. Yet, the authors present a reservation with respect to the generalizability of those findings since range restriction is in play, with the average intelligence of air force candidates well above average (Kratz et al., 2007). It is also worth noting that different versions of tests can create differently sized errors in the measurement, which will affect the size of an identified group difference, such as between genders. For instance, a meta-analysis by Giofrè et al. (2022) showed that older versions of the Weschler Intelligence Scale for Children (WISC) show larger gender differences compared to newer versions.

The primary aim of the present study is to make a distinct contribution to the knowledge of operational and cognitive tests from a gender perspective. Testing of *ab initio* pilot candidates– individuals with little or no flight experience–offers rich data to explore this since the candidates are not in any training (i.e., no training effects) and the test batteries contain different types of tests. The findings are relevant to all high-risk professions that use competence-based application procedures. The study will examine the gender differences between tests of operational and cognitive abilities, in terms of absolute scores, as well as the correlations between different tests (i.e., abilities) and compare the intercorrelations. Correlations between all tests will be explored and accounted for.

We hypothesize the following:

• Males will outperform women in manual and visual spatial tests, in line with previous findings (Halpern, 2013; Neubauer et al., 2005; Voyer et al., 1995)
• Females will outperform males in written tests of memory, in line with previous findings (Guiso et al., 2008; Neubauer et al., 2005)
• Males’ test profiles will be more heterogenous, in line with previous findings showing a seven percent larger standard deviation for men in tests of cognitive ability (Lubinski and Humphreys, 1990).

Our analysis will allow for a refined understanding of gender differences in cognitive and operational tests and the respective intercorrelations. This type of examination is necessary for tailoring of evaluation metrics, accounting for different strengths and weaknesses. We want to contribute to evidence-based selection and fairness in a sector where women are severely underrepresented.

## 2 Materials and methods

The test battery in focus in the present study has been used for selection of commercial pilots in Sweden since the first version was developed for Scandinavian Airlines by Scandinavian Institute of Aviation of Psychology (SIAP) in the 1950’s. (Trankell, 1959). A modern version of the test battery is employed presently in the selection of *ab initio* pilots applying for pilot training at Higher Vocational Education (HVE; in Swedish: Yrkeshögskolan), a post-secondary form of education that combines theoretical and practical studies in close cooperation with employers and industry (programs are offered in specific fields where there is an explicit demand for competence). Similar test batteries and test procedures are employed in many countries in both commercial aviation (e.g., Martinussen et al., 2022) and for Air Force selection (e.g., Carretta, 2011; Martinussen et al., 2022).

### 2.1 Participants and procedure

The data in the present study comes from 2,743 candidates aged between 18 and 48. 2,113 of the candidates applied for HVE at Lund University School of Aviation (LUSA) between 2009 and 2019. LUSA has provided HVE since 2009. The services of SIAP have been employed in the selection process every year. LUSA uses a stepwise elimination process. The candidates who were eliminated in earlier rounds of the process only performed the written tests (WT). Therefore, only 417 out of the 2,113 candidates completed the computer based Multitasking Test (MU) and the Computer Based Joystick Test of manual spatial ability (MSA). Data from the remaining 630 candidates included in the present study comes from General Aptitude Tests (GAT). Candidates in that process performed the WT, the MU and the MSA, without any elimination taking place in between, at SIAP between 2017 and 2019 to try and get a GAT certificate. A GAT certificate was needed to apply for pilot training– at LUSA or elsewhere– in Sweden during this period.

### 2.2 Test battery

#### 2.2.1 Written tests

The WTs were designed with the intention of capturing distinct subfactors.

• *Perceptual speed*. Find letter next to number in figure on busy page, write down the letter.
• *Memory retention*. Read a text about a flight, perform a 2-min distractor task, respond to multiple choice questions about text.
• *Spatial ability* (mental rotation). Folding a 3D figure mentally in accordance with a 2D pattern and choose between four alternatives as to which paper corresponds to figure.
• *Logical ability* (abstract problem-solving ability). Identify next in sequence of abstract images in an incomplete matrix.

#### 2.2.2 Multitasking test

The multitasking test (MU) was designed to capture certain operational abilities such as multitasking ability, perceptual speed, and the ability to divide attention. The test was performed on a Personal Computer. The test duration was 330 s and the candidates performed five tasks simultaneously. The tasks were: (i) to plot coordinates on a grid when a red lamp switched to green, (ii) to write down a number appearing in an arrow that moved up and down, (iii) to write down answers to mental arithmetic questions on a sheet of paper without prompt from the computer (iv), to state the position of an indicator verbally and point toward an up/down button on the screen whenever an indictor moved above or beneath a designated area, (v) to answer questions which appeared on the screen verbally (e.g., *What is the capital of Germany? What day was it 2 days ago? 13 times 11?*). The candidates were instructed to work with the different tasks to the same extent and to not neglect anything. The different tasks were intentionally set at different levels of auditory and visual signal strength. Part of the challenge for the candidates was to adequately prioritize tasks that did not automatically draw their attention. The difficulty of the individual tasks was set at a [lower] level where maximum problem-solving ability should not matter.

#### 2.2.3 Joystick test of manual spatial ability

The joystick test (MSA) was designed to capture spatial and manual ability in and of itself and under simultaneous increasing taxing of the working memory. The test was performed on a Personal Computer. The test duration was 300 s, and the candidates were instructed to simultaneously answer questions they were asked verbally (e.g., *Spell Stockholm backward! What is 13 times 11? Repeat the following numbers backward: 9381).* The candidates scored points through landing on a red or green target (which shifted during the test) by using a joystick while avoiding crashing into three moving obstacles.

### 2.3 Data analysis

Different versions of the tests, except for the joystick test of manual and spatial ability, were used at different years of testing. Test scores were standardized (*M* = 0, SD = 1) within each test version to account for possible differences in test difficulty. Each candidate received a score (when available) on the six tests included in the test battery that corresponded to their mean value on the items included in that test. Differences in test scores between female and male candidates were analyzed with independent samples *t*-tests. Associations between test scores were estimated with Pearson’s correlations and confidence intervals for the differences between correlations among female and male candidates were estimated by a test presented by (Zou, 2007). Analyzes were conducted with R 4.3.1 statistical software (R Core Team, 2023) employing the effsize (Torchiano, 2016), beanplot (Kampstra, 2008), and cocor (Diedenhofen and Musch, 2015) packages.

## 3 Results

**FIGURE 1:**
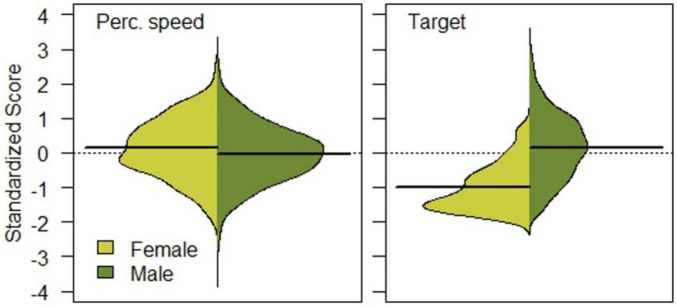
Distribution on perceptual speed **(left)** and manual spatial ability **(right)** among female and male applicants.

Of the 2,743 candidates, 2,325 were male (84.8%, mean age = 23.4 years, range = 18–50 years) and 417 were female (15.2%, mean age = 21.8 years, range = 18–39 years). Descriptive statistics separately for female and male applicants are presented in Table 1, as well as results from group comparisons. Female applicants had a significantly higher score on Perceptual speed. Male applicants, on the other hand, had a significantly higher score on the other five outcomes, with a notably large difference on the MSA-score. The significance of the differences was verified by non-parametric Mann-Whitney U-tests. Differences on Perceptual speed and MSA-score (under the test name Target) are illustrated in Figure 1.

**TABLE 1:** Descriptive statistics for and differences between female and male applicants on the study variables.

	Female		Male				
**Outcome**	***N***	**M (SD)**	***N***	**M (SD)**	**Cohen’s d (95% CI)**	***p*(T)**	***p*(U)**
Multitask*	146	−0.12 (0.65)	882	0.02 (0.60)	−0.22 (−0.40; −0.04)	0.014	0.018
Target**	155	−1.01 (0.65)	955	0.16 (0.95)	−1.28 (−1.46; −1.10)	<0.001	<0.001
Matrices	415	−0.08 (0.79)	2286	0.01 (0.85)	−0.11 (−0.21; 0.00)	0.043	0.037
Perc.speed	415	0.13 (0.84)	2288	−0.02 (0.81)	0.19 (0.08; 0.29)	<0.001	<0.001
Spatial	415	−0.24 (0.83)	2288	0.01 (0.83)	−0.31 (−0.41; −0.20)	<0.001	<0.001
Memory	360	−0.09 (0.86)	2000	0.02 (0.78)	−0.13 (−0.25; −0.02)	0.020	0.040

*p*(T) = *p*-value for an independent samples *t*-test; *p*(U) = *p*-value for a non-parametric Mann-Whitney U-test.

*Multitask = MU.

**Target = MSA.

**FIGURE 2:**
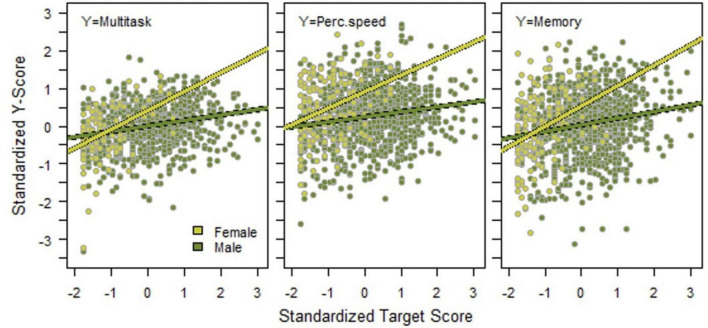
Associations between manual spatial ability and multitasking ability **(left)**, perceptual speed **(middle)**, and memory **(right)**, separately for female and male applicants.

Correlations between the outcome variables, separately for female and male applicants, are presented in Table 2. All correlations were statistically significant, both for female and male applicants and both when estimating Pearson and non-parametric Spearman correlations. Three correlations were significantly stronger among female compared with male applicants, namely between MT and JT [difference = 0.269 (95% CI: 0.114; 0.405)], between JT and Perceptual speed [difference = 0.186 (95% CI: 0.027; 0.332)], and between JT and Memory [difference = 0.171 (95% CI: 0.011; 0.317)]. These differences are illustrated in Figure 2. A high JT score was more predictive of a high score on MT, Perceptual speed, and Memory for female compared with male applicants (*p* < 0.002 for the JT score × sex interaction effect in all three cases). Among male applicants, a combination of a high score on JT and a low score on the other three variables was common. Thus, a stronger general factor is observed among women, the average female performance is more homogenous. As seen in Table 2 the correlations are stronger on average among women. This phenomenon is also observed in Figure 2.

**TABLE 2:** Pearson correlations between study variables for female (below diagonal) and male (above diagonal) applicants.

	1	2	3	4	5	6
1. Multitask	–	0.234	0.374	0.430	0.241	0.341
2. Target	0.503	–	0.253	0.165	0.287	0.197
3. Matrices	0.330	0.299	–	0.459	0.493	0.295
4. Perc.speed	0.519	0.351	0.399	–	0.401	0.311
5. Spatial	0.325	0.396	0.532	0.424	–	0.249
6. Memory	0.358	0.368	0.380	0.330	0.284	–

All correlations are highly significant (*p* < 0.001). Multitask = multitasking ability (MU); Target = manual spatial ability (MSA).

## 4 Discussion

This study set out to deliver on the mapping of gender differences and intercorrelations in operative and cognitive test scores. Our analyses showed that test scores differed significantly between the genders, as well as significantly in the correlations between tests within the genders, with a more heterogeneous test profile more common among men. Male applicants were stronger in several domains (mental and manual spatial ability, memory retention, abstract problem solving, multitasking ability), while the female candidates were stronger in perceptual speed. Regarding the size of the differences identified in our study: According to Cohen’s guidelines, gender differences were small on Matrices, Perceptual Speed, and Memory, medium on Multitask and Spatial Ability, and large on Target, respectively. All effects fulfilled Hyde (2005) criterion of non-trivial gender differences (*d* ≥ 0.10). Moreover, differences were larger than a meta-analytically estimated overall gender difference on spatial ability (*d* = −0.37) (Voyer et al., 1995) on Target but smaller on the other five tests.

The correlations between multitasking ability and manual spatial ability, and between mental spatial ability, were significantly stronger among female candidates. A strong performance in manual spatial ability was more predictive of a strong performance in multitasking, perceptual speed, and memory retention, among females. The male candidates were significantly stronger in manual spatial ability than the female candidates. However, a strong manual spatial performance on its own was less predictive for an overall satisfactory score for a male. It was more common to be strong manually but lack the other skills thought to be predictive for success in flight school and beyond.

Our findings agree with earlier observations which found that men perform better in tasks involving mental rotation (Neubauer et al., 2005) and spatial ability (Halpern, 2013); which in turn has been put forward to explain a stronger performance in multitasking tests (Mäntylä, 2013). Our analysis showed that men performed stronger in the memory task. This outcome is contrary to that of for instance Herlitz and Rehnman (2008). What could explain this discrepancy? A test-related worry might have affected the females’ performance, as per the findings of Beilock (2008). According to processing efficiency theory, worrisome thoughts interfere with working memory (Eysenck and Calvo, 1992). This interference has been found to be more prominent among females (Ganley and Vasilyeva, 2014). Pilot testing is an especially male-dominated context; thus this test-related worry, potentially heightened by stereotype threat, may have been [even] stronger than in many other test contexts. The discrepancy may also be partially due to the nature of the material in the memory task, as identified in the above-mentioned meta-analyses by Asperholm et al. (2019). The nature of the memory task in our study was to a large degree to memorize numbers and times, and males have previously been found to be stronger in numerical tasks (e.g., Guiso et al., 2008; Weber et al., 2014).

With respect to males outperforming females overall, if test–related worry affected working memory performance, most tests scores would be affected (Beilock et al., 2007). The only subtest in the present study where females outperformed males were tests of perceptual speed– a test dependent on general intelligence, but not active manipulation of the working memory– thus, a test less affected by potential concurrent worry.

It was more common for men to perform strongly in the test of manual spatial ability and poorly in other tests in the present study. In other words, to demonstrate a strong spatial ability without being strong in terms of general cognitive ability and/or multitasking ability. Why is the average male performance more heterogenous? Evidently, multitasking ability is partially explained by general cognitive ability (Redick et al., 2016), yet may be hindered by relative weaknesses in subfactors, such as for instance perceptual speed (where the women in the present study performed better) and was not associated with a strong manual and spatial performance. Conversely, if a candidate demonstrated a strong perceptual speed, they tended to perform well in the other written tests (note: performed one at a time), while it was no guarantee for a good score in neither multitasking ability nor spatial ability. In the present study, men had a higher overall score. One reason for this may be that the distribution of IQ-points is different in men and women; more men are found among the lowest and highest scorers, with previous finding showing a 7 percent larger standard deviation for males (Lubinski and Humphreys, 1990). People applying for flight school are likely to be above a certain minimum level of cognitive functioning, thus excluding the low-performing males. The sample in the present study is pre-selected, and this pre-selection process may look different between the genders. Since the conception of the tests, cognitive functioning, and the ability to multitask are constructs conceived to be of relevance to prevent accidents from happening (Trankell, 1959). From a long-term risk perspective, the threshold level of cognitive ability likely also indirectly screens for risk-taking behavior (Gottfredson, 2004) and psychological health (Deary and Batty, 2007), however the association between cognitive ability and depressive symptoms doesn’t remain constant as people age (Lager et al., 2017).

In a broader sense, it is important to note that the factors potentially affecting the relative gender proportions in the aviation industry likely are at play in the present study. The differences in test scores in a male dominated field such as aviation may to some extent depend on stereotype threat. Activating stereotypes damages task performance (Stangor et al., 1998) which can be a factor negatively affecting women in aviation selection. Also, in a test situation in general, men estimate their own intelligence higher than women (e.g., Furnham and Rawles, 1999). We found that one general factor explained more of the variance among women than among men. Why is this? Why is there a stronger association between manual spatial ability and multitasking ability for women? Do they need a stronger overall ability to work fast to have “attention to spare”? That partial explanation is supported by the significantly stronger association between spatial ability and perceptual speed for women. If high-performing women can do things quickly, they can switch between tasks and perform strongly in multitasking. Conversely, a man may demonstrate a relatively strong performance partially because of how little of the attention span that is taxed by steering the joystick. Similarly, when the female candidates demonstrated stronger memory performance, they tended to perform better in the manual spatial test. This may to some extent be explained by their performance not being compromised by the simultaneous working memory tasks. They had more “focus to spare,” perhaps. In future research efforts it may be of interest to also assess the difficulty of task that the candidates manage to complete simultaneously since it would provide even more nuanced data on the interplay between spatial ability, working memory and simultaneous ability.

It is notable from a risk perspective, in the specific case of commercial aviation, that the gender differences observed in this study fit with analyses of accidents. However, this relationship must be considered with caution since accidents are a multifactorial and complex phenomenon. Male pilots have previously been found to be more likely to have accidents due to inattention and/or poor planning, while female pilots have been found to be more likely to have accidents due to mishandling of the aircraft (Baker et al., 2010). What is more, it has been found that female pilots have significantly more accidents than men at lower levels of experience, and significantly fewer accidents at higher levels of experience (Walton and Politano, 2016). In other words, pilots get better at handling the aircraft with experience, and this reduces the risk of accidents among female pilots more because of baseline values. At least if a [general] potential is there. Fittingly, our analyses showed that overall, relatively strong female candidates often performed quite poorly in terms of manual spatial ability. In male candidates, we observed that some performed strongly in manual spatial ability while demonstrating a relative inattentiveness/inability to divide attention, in the multitasking test. Again, caution must be exercised when connecting the dots between our findings from selection tests and risks long term. However, identifying relevant group differences is a part of the puzzle.

There are suggestions that accident differences partially are effects of gender with men being more impulsive to take risks (Baker et al., 2010; Jonas, 2001). This may partially be explained by the possibility of having a strong spatial and manual ability without having a higher intelligence, which is associated with less accident-related risk-taking behavior (Gottfredson, 2004). It has previously been established that there are gender differences in risk-taking behavior, with men taking more risks (e.g., Byrnes et al., 1999). However, an understanding of risk-related and/or impulsive behavior can be conceptualized and framed in numerous ways. For instance, research using cognitive reflection tests (CRT) (see Frederick, 2005), has demonstrated that men were better at resisting giving an incorrect–but intuitive– answer (Cueva et al., 2016; Frederick, 2005). Evidently, accidents in aviation also depend on other factors than the individual. For instance, they have been identified by the pilots themselves to largely depend on working conditions (Melin et al., 2018), while the perspective of what constitutes ecologically valid data in terms of risks differs between professions within aviation (Lager and Melin, 2022).

In the specific case of aviation, training has been found to get both men and women to reach comparable levels of performance for their relative weaknesses. Simply put, gender differences decrease through adequate training (Neubauer et al., 2010). This has immediate implications for selection. It is possible that potentially strong candidates are excluded too early in the process based on absolute level of performance in a sub test prior to training. Research on pilot training shows that men and women tend to reach a similar level of flight performance after the same length of training periods and number of simulator exercises (Bauer et al., 2019). If training did not work for both genders, there would be differences in accident rates.

In conclusion, the findings of the present study highlight the need to properly frame the interpretation of female and male candidates’ test scores. In turn, recognizing group differences enables addressing of significant gender–related roadblocks. These perspectives– or angles to address unfairness and evidence-based selection– are not opposing views but rather complementary ones. An important issue for future research is to link test performance–in both operational and cognitive test– at the time of selection, with ecologically valid outcomes, during and after training. All stakeholders likely agree that the aviation industry, and other high-consequence industries, must be staffed by competent people, selected on potential and competence. This starts with valid selection, not myths and stereotypes.

## Data Availability

The raw data supporting the conclusions of this article will be made available by the authors, without undue reservation.
